# Biomimetic carriers mimicking leukocyte plasma membrane to increase tumor vasculature permeability

**DOI:** 10.1038/srep34422

**Published:** 2016-10-05

**Authors:** R. Palomba, A. Parodi, M. Evangelopoulos, S. Acciardo, C. Corbo, E. de Rosa, I. K. Yazdi, S. Scaria, R. Molinaro, N. E. Toledano Furman, J. You, M. Ferrari, F. Salvatore, E. Tasciotti

**Affiliations:** 1 Center for Biomimetic Medicine, Houston Methodist Research Institute, 6670 Bertner Avenue, Houston, Texas 77030, USA; 2 Fondazione SDN, Via Emanuele Gianturco 113, 80143 Naples, Italy; 3 Department of Mechanical and Aerospace Engineering, Politecnico di Torino, Corso Duca degli Abruzzi 24, 10129 Turin, Italy; 4 Department of Nanomedicine, Houston Methodist Research Institute, 6670 Bertner Avenue, Houston, Texas 77030, USA; 5 CEINGE-Biotecnologie Avanzate S.c.a.r.l, Via Gaetano Salvatore 486, 80145 Naples, Italy; 6 Department of Orthopedics, Houston Methodist Research Institute, 6670 Bertner Avenue, Houston, Texas 77030, USA

## Abstract

Recent advances in the field of nanomedicine have demonstrated that biomimicry can further improve targeting properties of current nanotechnologies while simultaneously enable carriers with a biological identity to better interact with the biological environment. Immune cells for example employ membrane proteins to target inflamed vasculature, locally increase vascular permeability, and extravasate across inflamed endothelium. Inspired by the physiology of immune cells, we recently developed a procedure to transfer leukocyte membranes onto nanoporous silicon particles (NPS), yielding Leukolike Vectors (LLV). LLV are composed of a surface coating containing multiple receptors that are critical in the cross-talk with the endothelium, mediating cellular accumulation in the tumor microenvironment while decreasing vascular barrier function. We previously demonstrated that lymphocyte function-associated antigen (LFA-1) transferred onto LLV was able to trigger the clustering of intercellular adhesion molecule 1 (ICAM-1) on endothelial cells. Herein, we provide a more comprehensive analysis of the working mechanism of LLV *in vitro* in activating this pathway and *in vivo* in enhancing vascular permeability. Our results suggest the biological activity of the leukocyte membrane can be retained upon transplant onto NPS and is critical in providing the particles with complex biological functions towards tumor vasculature.

The specific targeting of cancer lesion remains the primary goals of nanomedicine applied to oncological disease and represents a promising opportunity to increase poor cancer patient survival^1^,^2^. Over the past decades, nanomedicine has provided several delivery platforms demonstrated to enhance chemotherapeutic delivery^3^,^4^,^5^, however, current results are still unsatisfactory^6^. As demonstrated by our group^2^,^7^,^8^ and others^9^, a significant accumulation in the cancer lesions is hampered by several biological barriers (e.g., mononuclear phagocytic system, tumor-associated vasculature, tumor extracellular matrix, and cellular membrane) standing between the point of administration and the pathological site. The ideal treatment should be able to overcome each of these barriers in a sequential manner to reach its intended site^10^. The successful negotiation of tumor-associated vasculature represents one the greatest challenges in improving the effectiveness of current treatments and diagnostic tools^8^,^11^,^12^.

Previously, nanocarrier accumulation relied on exploiting the superior permeability of tumor vasculature^13^, a phenomenon commonly referred to as the enhanced permeability and retention (EPR) effect. Further understanding of the ultrastructure and transport that occurs in cancer lesions allowed for the rational development of carriers that specifically target diseased tissue by exploiting lesion-specific transport oncophysics^14^. On the other hand, a better understanding of the biological features characterizing tumor blood vessels^15^ highlighted the possibility to design carriers with biological properties^8^,^16^,^17^, prompting a deeper investigation into alternative vector-associated modifications^18^ and tumor characteristics^19^. In particular, cancer associated inflammation^20^ and tumor vasculature^21^ provides several opportunities to develop targeted therapies by leveraging the adhesive proteins over-expressed on inflamed vessels^22^.

We recently demonstrated a technique for the functionalization of the surface of nanoporous silicon particles (NPS)^8^ with purified leukocyte membranes. These NPS were previously shown to be biocompatible^23^, degradable^24^, and able to be rationally designed in order to cross a multiplicity of sequential biological barriers to attain preferential concentration at desired target cancer locations^2^,^12^. These NPS formed the basis for multi-stage vectors^25^ and injectable nanoparticle generators for the cure of visceral metastases in triple-negative breast cancer^26^. The functionalization of NPS with purified leukocyte membrane was demonstrated on select variants of the NPS platforms^8^, yielding leukolike vectors (LLV), which displayed properties similar to their leukocyte source while preserving some favorable properties of NPS (e.g. drug loading and release, margination) on those select variants. Specifically, LLV were demonstrated to be successfully functionalized with more than 150 leukocyte membrane-associated proteins, including adhesive surface proteins involved in leukocyte diapedesis^27^ and were shown to efficiently interact with intercellular adhesion molecule-1 (ICAM-1) inducing its clustering^8^. ICAM-1 is overexpressed in tumor-associated vasculature and is involved in leukocyte adhesion and endothelial reorganization^28^. This process is critical in mediating vascular permeability as a result of decreased expression of endothelial intercellular junctions at the endothelial cell border^29^, thereby favoring immune cell infiltration^30^,^31^. In this work, we confirmed that the cell membrane applied on the surface of synthetic NPS remained functional in triggering the biomolecular events that culminate in increased vascular permeability. In addition, we demonstrated that the coating maintained its biological properties also *in vivo,* favoring LLV firm adhesion on tumor-associated vasculature and resulting in increased perfusion of small molecules into the subendothelial space. More so, we definitively validated that specific biological activities that characterize the surface of leukocytes can be transferred onto synthetic carriers, providing them with a biological identity and favoring their molecular interaction with vascular tissue both *in vitro* and *in vivo*.

## Results and Discussion

### Surface characterization of Leukolike Vectors

**Figure 1:**
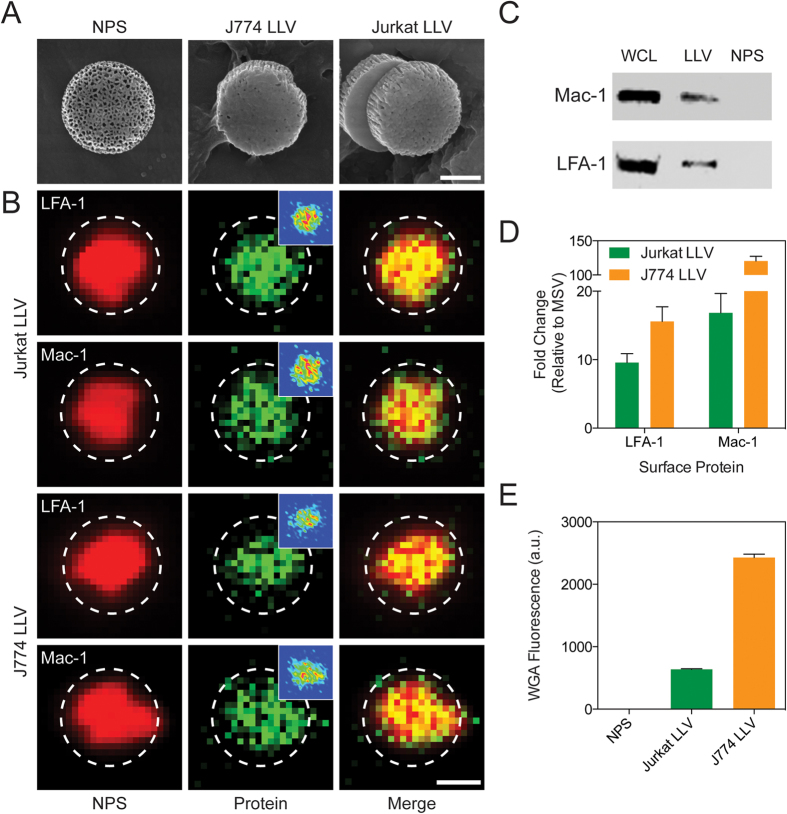
Particle Characterization. (**A**) SEM images of uncoated particles (NPS) and particles coated with cellular membrane derived from murine macrophages (J774 LLV) and human T-cells (Jurkat LLV). (**B**) Fluorescent microscope images of LLV-modified with Alexa Fluor 555 (red, first column) and immunofluorescent staining of Jurkat LLV and J774 LLV for surface markers LFA-1 and Mac-1 (green, second column) and merged (third column). (**C**) Western blot analysis of leukocyte adhesion molecules successfully transferred on LLV (WCL: whole cell lysate). (**D**) Flow cytometry analysis of particles revealing the presence of LFA-1 and Mac-1. (**E**) Flow cytometry analysis of the particles stained for wheat germ agglutinin. The data are plotted as the mean ± s.d.

LLV were assembled using 1 μm discoidal NPS as previously reported^8^. Briefly, LLV were fabricated using cellular membranes purified from human T-cells (Jurkat) or murine macrophages (J774) to minimize reactivity^32^ and closely mimic the biological vasculature activity that will be tested *in vitro* (i.e., human) and *in vivo* (i.e., murine), respectively. The membrane coating on the NPS surface was stabilized using electrostatic interactions between the negatively charged cellular membrane and the positively charged NPS, previously modified with (3-Aminopropyl) triethoxysilane (APTES). Scanning electron microscope micrographs revealed uniform membrane coating on the LLV surface with minimal exposure of the underlying nanopores (Fig. 1A). Zeta potential analysis demonstrated a positive charge after functionalization with APTES, while coating the NPS core with cellular membrane proteins resulted in a negative surface charge for both LLV formulations (Supplementary Fig. S1). This result was in accordance with the negative surface charge of native leukocytes^33^.

Next, fluorescent microscopy revealed the homogenous distribution of lymphocyte function-associated antigen 1 (LFA-1) and macrophage-1 antigen (Mac-1) adhesive proteins on the particle surface for both Jurkat LLV and J774 LLV (Fig. 1B). Their presence was further validated through western blot analysis and flow cytometry (Fig. 1C,D). These proteins have previously been shown to be fundamental in the activation of ICAM-1 expression on endothelial cells^28^,^34^. To assess their role in the adhesion of LLV towards an inflamed endothelium, human umbilical vein endothelial cells (HUVEC) were treated with anti-LFA-1 LLV and anti-Mac-1 LLV under physiological flow conditions and compared to LLV (Supplementary Fig. S2). Our data revealed that compared to LLV, both anti-LFA-1 and anti-Mac-1 LLV resulted in decreased adhesion to the endothelial cells, confirming that both of these proteins participate in the interaction with inflamed vasculature (Supplementary Fig. S2A). Furthermore, it was observed that the blocking of LFA-1 alone resulted in a significant inhibition of particle accumulation relative to Mac-1-blocked LLV (Supplementary Fig. S2B,C). A similar phenomenon was observed *in vivo* using intravital microscopy by administering LLV, anti-LFA-1 LLV, and anti-Mac-1 LLV to BALB/c 4T1 breast cancer tumor-bearing mice. Blocking LFA-1 and Mac-1 both demonstrated a decrease in particle accumulation at tumor vasculature, with LFA-1 representing a significant decrease compared to LLV (Supplementary Fig. S3).

In addition, flow cytometry revealed post-translational modifications of adhesive proteins were maintained on the LLV surface as demonstrated by wheat germ agglutinin staining (Fig. 1E). The addition of the coating was also found to display minimal changes in particle size as demonstrated by dynamic light scattering (Supplementary Fig. S4) and SEM images revealed a lack of particle aggregation following coating (Supplementary Fig. S5). This data provides a general physical, chemical, and biological characterization of the system, exhibiting the successful transfer of biological material onto synthetic particles and indicating the presence of the machinery necessary to adhere and activate the ICAM-1 pathway in inflamed endothelium.

### ICAM-1 pathway activation

**Figure 2:**
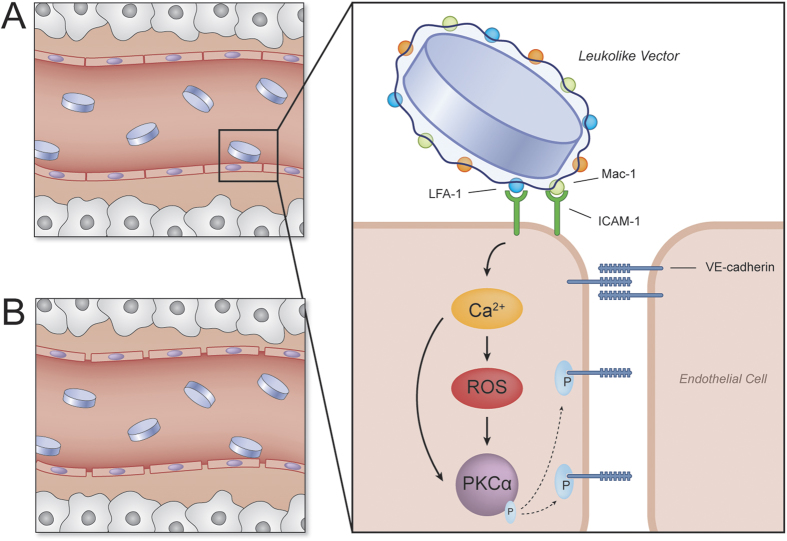
ICAM-1 pathway activation schematic. Activation of ICAM-1 pathway by LLV: (**A**) LLV adhere to inflamed endothelium interacting with ICAM-1 through adhesive receptors LFA-1 and Mac-1 (see inset). This interaction is efficient in activating the ICAM-1 pathway. Subsequently, ICAM-1 pathway activation results in an increase in intracellular calcium and ROS concentrations, resulting in an independent activation of PKCα. PKCα increases lead to the phosphorylation of VE-cadherin, resulting in the disassembly of VE-cadherin and protein displacement. (**B**) Following protein displacement of VE-cadherin, gaps between endothelial cells form, leading to an increase in vascular permeability and payload transport into the extracellular matrix.

**Figure 3:**
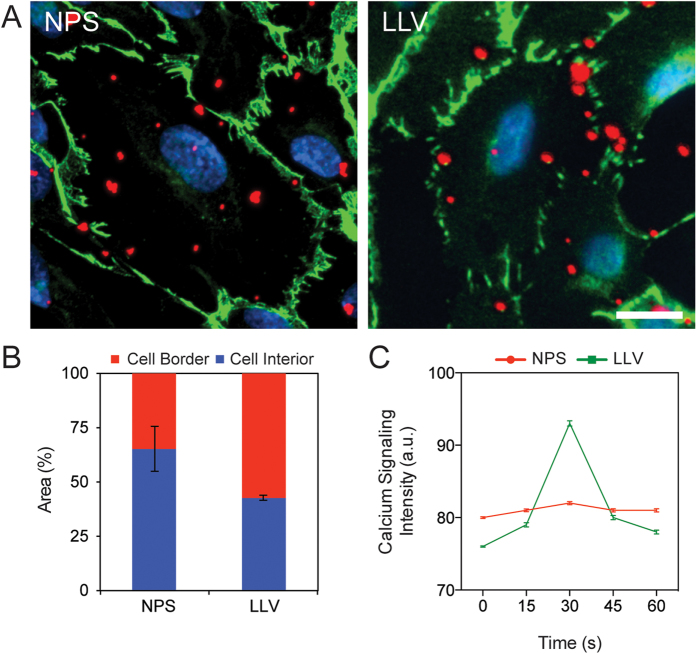
Adhesion proprieties and effect on calcium signaling in inflamed endothelium. (**A**) Representative images of NPS and LLV (red) adhered on endothelial cells following a brief flow of particles to discriminate between particles bound on the cell border and cell interior. VE-Cadherin junctions of endothelial cells were labeled with an anti-VE-cadherin antibody (green) and nuclei were stained with DAPI (blue) (scale bar: 25 μm). (**B**) Graph representing differential LLV and NPS distribution on cell border or interior. (**C**) Calcium signaling following particle flow was assessed through a Fluo3 AM staining monitored in live microscopy. The data are plotted as the mean ± s.d.

Previously, we demonstrated that LLV is capable of inducing ICAM-1^8^ clustering. Herein, we focused our attention to assess if this phenomenon was effectively followed by the activation of ICAM-1 pathway and determine its implication in terms of vascular permeability (Fig. 2). All experiments were performed under flow on an inflamed endothelial monolayer developed using HUVEC activated with tumor necrosis factor alpha (TNF-α) treatment for 24 h. This model has been extensively used to investigate particle adhesion in flow dynamics^35^,^36^. In these experimental conditions, endothelial cells overexpress ICAM-1, as shown in (Supplementary Fig. S6)^37^. Following a 10 min. perfusion of particles at a rate of 0.1 dyn/cm^2^, LLV preferentially accumulated at the cell-cell border, while NPS distributed more homogeneously on the surface of the cells (Fig. 3A). This finding suggests that the LLV preferentially adhered at cell edges and revealed that 23% more LLV localized at the cell borders when compared to NPS (Fig. 3B). Additionally, literature and our previous work exhibited that the border of inflamed endothelial cells is predominantly enriched with ICAM-1 to engage surface interactions with circulating leukocytes^8^,^38^. In nature, the activation of the ICAM-1 pathway by leukocytes induces an increase in the intracellular concentration of Ca^2+^ 39. To measure the changes in Ca^2+^ production following treatment with LLV, a combination of fluorometric analysis and live microscopy was used on a HUVEC monolayer. Increases in the cytoplasmic levels of Ca^2+^ were observed as quickly as 15 s following interaction of LLV with inflamed endothelium (Fig. 3C). This finding corroborates results obtained by previous literature describing leukocyte extravasation^40^.

**Figure 4:**
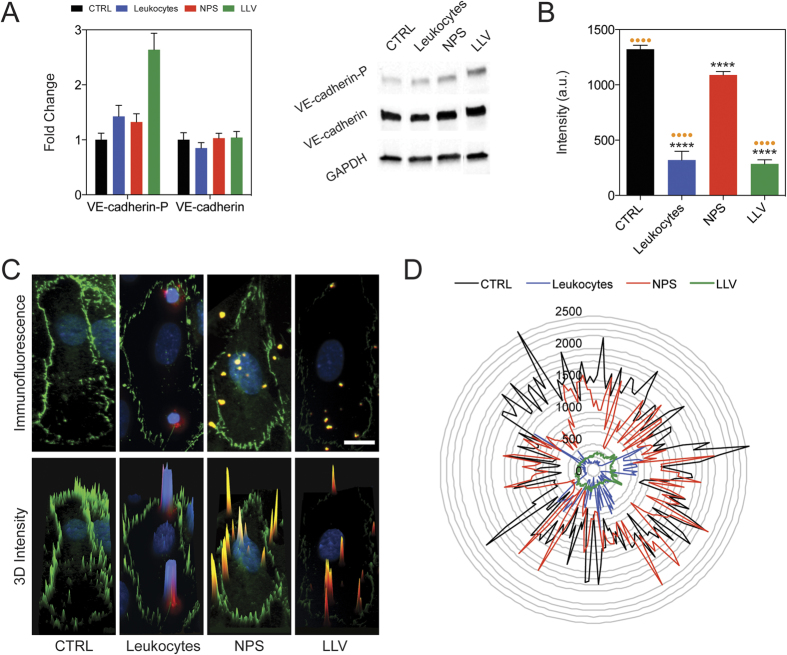
ICAM1 pathway activation. (**A**) Western blot analysis of Ve-cadherin-P and VE-cadherin 15 min. following particles and leukocytes treatment. Expanded Western Blot images are displayed in Supplementary Figure S7. (**B**) Quantitative analysis of VE-cadherin expression on cell border of TNFα-activated HUVEC treated with a flow of leukocytes or particles. Data were obtained by immunofluorescence. Fluorescence intensity was measured along the perimeter of HUVEC per condition (n = 15). (**C**) Immunofluorescence images and tri-dimensional fluorescence intensity profile (3D Intensity) showing single TNFα-activated HUVEC. (**D**) Intensity profiles of the cell perimeter of single TNFα-activated HUVEC plotted in polar coordinates. For B, C and D images the analyses were performed on untreated HUVEC (CTRL) and on HUVEC treated with Jurkat cells (Leukocytes), uncoated particles (NPS), and coated particles (LLV). The data are plotted as the mean ± s.d. Statistical analysis was performed using a one-way ANOVA with a Turkey post-test. Asterisks denote significance relative to CTRL. Dots denote significance relative to NPS. ****P < 0.0001.

Furthermore, ICAM-1 pathway activation involves the phosphorylation of protein kinase C alpha (PKCα) that, in turn, phosphorylates VE-cadherin^41^,^42^, leading to its membrane displacement and the partial disruption of the endothelial intercellular junction. VE-cadherin is responsible for maintaining the endothelial monolayer’s continuity and barrier function^29^,^43^. Western blot analysis was used to assess the phosphorylation levels of VE-cadherin and PKCα on TNFα-activated HUVEC following treatment with LLV or NPS, while an untreated control and leukocytes (i.e., Jurkat T-cells) were used as negative and positive control, respectively. The analysis revealed that VE-cadherin phosphorylated protein (VE-cadherin-P) was 2.5-fold higher in LLV-treated HUVEC than in untreated cells, while the level of VE-cadherin-P slightly increased in NPS-treated cells, maintaining basal levels of phosphorylation similar to the controls (Fig. 4A, Supplementary Fig. S7). On the other hand, VE-cadherin-P expression was 1.5-fold higher in leukocyte-treated HUVEC than in controls (Fig. 4A), indicating that LLV retained the critical biological determinants necessary to induce VE-cadherin phosphorylation, while no significant changes occurred in total VE-cadherin protein expression after treatment. Similarly compared to an untreated control, endothelial cells treated with LLV and leukocytes increased their basal expression of PKCα phosphorylated protein (PKCα-P) (Supplementary Fig. S8). The phosphorylation of these two important mediators represents a critical step in the functional down-regulation of VE-cadherin as it determines its cytoplasmic displacement from the edge of endothelial cells.

VE-cadherin displacement from the membrane has previously been reported as an effect produced by leukocytes on endothelial cells after activation of the ICAM-1 pathway^31^. This phenomenon was evaluated through fluorescence microscopy following particle flow, using similar experimental settings as described above. Under conditions that mimic capillary flow *in vitro*, inflamed HUVEC monolayers were exposed for 30 min. to leukocytes, NPS, or LLV (Fig. 4B). VE-cadherin expression along the cell perimeter was then analyzed by immunofluorescence. In comparison to untreated and NPS-treated cells, VE-cadherin expression decreased significantly (p < 0.0001) in the group treated with LLV and leukocytes (p < 0.0001) (Fig. 4B).

Representative immunofluorescence images acquired following treatment are shown in Fig. 4C. This data can also be observed in a tridimensional fluorescent analysis on the acquired images (Fig. 4C) and by plotting the fluorescence intensity profile of the cell perimeter in polar coordinates (Fig. 4D). Conversely, VE-cadherin was only slightly decreased in non-inflamed endothelium after exposure to LLV (Supplementary Figs S9–S11). Collectively, this data confirms the specificity of our biomimetic delivery platform towards inflamed endothelia and its ability to actively trigger the ICAM-1 pathway. In particular, the proteolipid coating applied on the surface of the particles was effective in favoring VE-Cadherin phosphorylation and displacement, inhibiting the intercellular connections between the cells composing the monolayer.

### LLV targeting and bioactivity *in vivo*

**Figure 5:**
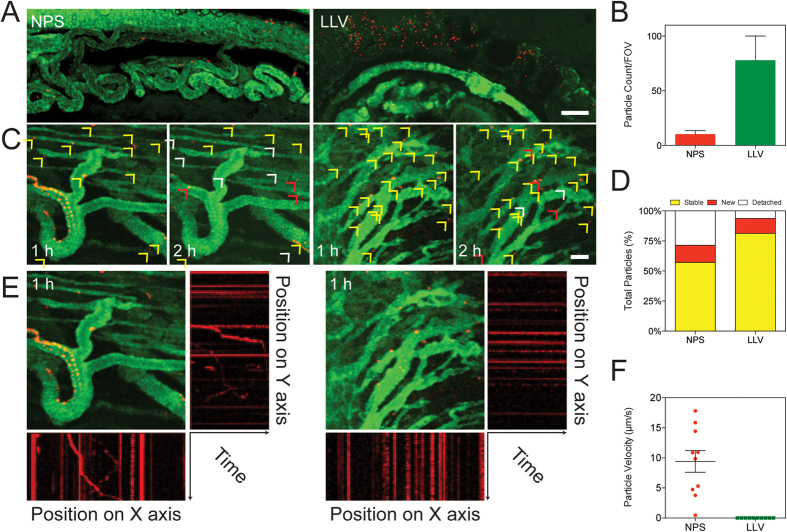
Intravital microscopy analysis of LLV tumor endothelium targeting and binding stability. (**A**) Intravital microscopy images of orthotopic 4T1 tumor following treatment with NPS and LLV (scale bar: 100 μm). (**B**) Quantification of particles bound to tumor vasculature, count was performed on same area fraction. (**C**) Intravital microscope images portraying binding stability of LLV and NPS on tumor endothelium at 1 and 2 h created by merging together consecutive frames obtained from 20 sec movies (scale bar: 50 μm). Arrows indicate new (red), stable (yellow), or detached (white) particles. (**D**) Quantification of binding stability determined from intravital microscope images. (**E**) Particle motion analysis in tumor vasculature. Plotting X or Y particles position as a function of time, firmly bound particle events appear as straight lines while moving particle events appear as askew lines. (**F**) In the Graph we report the registered velocity of moving NPS particles compared to LLV particles which appears all in steady state. The data are plotted as the mean ± s.d.

We next investigated the advantages of LLV for targeting tumor-associated vasculature and in increasing its permeability in an orthotopic 4T1 breast cancer tumor model. Ten days after tumor establishment, mice were treated with either NPS or LLV, followed by a single injection of a 70 kDa fluorescein isothiocyanate-dextran tracer (3% w/v) to define tumor vasculature. The membrane coating applied on the LLV increased the targeting potential when compared with NPS (Fig. 5A,B), concurring with previously published results obtained in a melanoma model^8^. In an attempt to shed light on the spatiotemporal mechanics of LLV interaction with tumor endothelium, we carried out a time-dependent evaluation of particle binding to the endothelium 1 and 2 h following particle injection. Specifically, random sections of the tumor vasculature were assessed for the ability of particles to: 1) establish new binding events (in red), 2) firmly adhere to the tumor vasculature (in yellow), and 3) detach from the vessel wall (in white) (Fig. 5C,D). The LLV and NPS showed similar properties in interacting with the tumor-associated vasculature, likely a result of the particle shape strategically designed to favor margination in the tumor capillaries^44^,^45^. More so, the application of the leukocyte coating onto the NPS resulted in a 2.16-fold reduction in LLV detachment (Fig. 5D). This suggests that the adhesion proteins on the LLV surface played an active role in the adhesion to the vessel wall and that key leukocyte proteins remain functional even after contact with the biological surface.

To further investigate the ability of LLV to firmly adhere to an inflamed tumor vessel wall *in vivo*, we developed a novel analytical tool by merging consecutive frames obtained by intravital microscopy (IVM) movies into one image. Thus, the time course experiment was resolved into a series of single images in which the fluorescence of the LLV and NPS indicated the particle positions in the different frames (Fig. 5E). When these positions were projected onto a Cartesian coordinate system as a function of time, firmly bound LLV appeared as a straight horizontal or vertical line according to their respective X and Y coordinates, while NPS appeared as slanted lines, indicating reduced adhesion. Furthermore, slope calculations demonstrated the average velocity for traveling NPS remained at 9.8 μm/s while LLV remained at 0 μm/s, suggesting stable adhesion (Fig. 5F).

**Figure 6:**
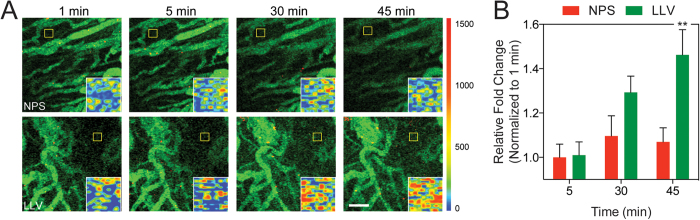
Intravital microscopy analysis of 70 kDa dextran extravasation. (**A**) Tumor vasculature images of mice administered with dextran following NPS and LLV injection, (scale bar: 100 μm). Images were acquired over 45 min. Insets represent a heat map of yellow box to highlight dextran extravasation. (**B**) Quantitative analysis on relative fold change of dextran penetration into the subendothelial space. The data are plotted as the mean ± s.d. Statistical analysis was performed using a two-way ANOVA with a Bonferroni post-test. **P < 0.01.

To gain further insights into the permeability of tumor vasculature following exposure with LLV, we measured the time-dependent extravasation of the intravenously administered fluorescent tracer 70 kDa dextran (Fig. 6A). Using IVM, movie frames of the sub-endothelial tumor space were collected 1, 5, 30 and 45 min. after dextran administration. IVM images showed a linear increase in dextran extravasation in mice treated with LLV and NPS. However, 45 min. following treatment, dextran extravasation was more than 35% higher in LLV-treated mice compared to NPS-treated (Fig. 6B). This phenomenon was further confirmed by developing an intensity map of representative sections of the sub-endothelial space (Fig. 6A, inset) where the color code indicated a prominent extravasation of the fluorescent dye after 45 min. To analyze the penetration potential of dextran into the subendothelial space, a subsection beginning at the vessel and covering the subendothelial space was analyzed. This confirmed that LLV modulated the endothelial barrier, allowing the dextran to penetrate deeper into the subendothelial space and serves as a representation of how therapeutics (i.e., particles >70 kDa) can penetrate into the subendothelial space following LLV adhesion (Supplementary Fig. S12). In addition, the preferential accumulation of LLV at the tumor vasculature can further benefit from the working mechanism of NPS and deliver larger therapeutic agents through the degradation of the silicon core^8^,^25^,^46^. Together, this data demonstrates that the leukocyte membrane coating enhances diffusion through the tumor vasculature *in vivo* by engaging specific surface interactions with the endothelial cells.

## Conclusion

The last decade has seen the emergence of biomimetic strategies^47^ as promising alternatives to drug delivery platforms based on synthetic materials and the exploitation of the EPR effect^48^,^49^. LLV have been fabricated based on the fusion of synthetic, modifiable NPS and purified leukocyte cell membrane. This coating has previously been demonstrated as retaining the properties portrayed by NPS, as demonstrated by the loading and release of model payloads (i.e., Doxorubicin and Albumin)^8^. In this work, we further validated that the coating does not interfere with the margination properties of NPS but rather enhanced the particle interaction with tumor blood vessels, providing a synergistic effect that results in superior targeting and firm adhesion. Additionally, we demonstrate that the coating could molecularly interact with the surface of the cell. Specifically, purified leukocyte plasma membranes grafted on the NPS surface efficiently activate the endothelial receptor ICAM-1 pathway, resulting in increased vascular permeability through the phosphorylation of VE-cadherin. Furthermore, *in vivo* studies demonstrated that this approach enhanced the targeting properties, promoted firm adhesion to the tumor vasculature, and increased tumor perfusion. This work provides further confirmation of the potentialities in implementing synthetic materials with biological components for overcoming the current limitation in nanocarrier fabrication^50^ and improve the treatment of diseases characterized by leukocyte infiltration. From this work, we can conclude that the cell membrane isolated and applied onto NPS at least partially preserves its biological activity. Although the translation properties of this approach require further characterization, particularly in defining the specific mechanistic properties of LLV bioactivity, we demonstrate that the biomolecular properties remain functional, highlighting a suitable alternative approach to current nanocarrier design and a significant advance in the development of future biomimetic technologies^49^.

## Materials and Methods

### Leukolike vector fabrication

NPS were fabricated at the Microelectronics Research Center at The University of Texas at Austin (Austin, TX, USA), as reported elsewhere^51^. APTES-conjugation was performed by mixing oxidized NPS in a solution containing 2% APTES (Sigma–Aldrich, St. Louis, MO, USA) and 5% MilliQ water in isopropyl alcohol and mixed under continuous and constant agitation for 2 h at 35 °C. After incubation, particles were washed three times in isopropanol and stored in IPA at 4 °C. Fluorescent labeling of NPS was achieved by mixing them in a 100 mM triethanolamine (in DMSO) solution containing AlexaFluor 488 or 555 (1 mg/mL, Life Technologies) for 2 h at room temperature under brief agitation. NPS were then washed to remove free dye and stored at 4 °C in isopropyl alcohol.

The LLV were fabricated following protocols previous established by our group^8^. Cell membranes were isolated by brief homogenization in a Dounce homogenizer and spun down at 500× g for 10 min at 4 °C. The supernatant was collected and pooled after three additional homogenization steps. The pooled supernatant was placed on a discontinuous sucrose gradient (55-40-30% w/v sucrose) and centrifuged at 28,000× g for 45 min. The membrane at the 30–40 interface was collected and washed again in 150 mM NaCl solution. It was then mixed with APTES-conjugated NPS using a 1.5:1 (membrane:particle) mass ratio and incubated overnight under continuous rotation at 4 °C. Unbound membranes were then washed using 150 mM NaCl solution by centrifugation using a setting of 750× g for 10 min. Dynamic light scatter and zeta potential were performed by suspending 10^7^ particles in MilliQ water and measured for the particle size using a Zetasizer Nano ZS (Malvern, Malvern, UK). The sample was then placed into a disposable folded capillary cell and measured for the zeta potential. Jurkat cell membranes were used for *in vitro* studies while J774 cell membranes were used for *in vivo* studies.

### Flow cytometry

Surface proteins were quantified by mixing 5 × 10^6^ particles in a FACS buffer solution (1% bovine serum albumin, BSA) blocking solution for 30 min. Next, particles were washed and allowed to mix with FITC Rat Anti-Mouse CD11a (LFA-1) or Alexa Fluor® 488 Rat Anti-Mouse CD11b (Becton Dickinson, Houston, TX, USA) suspended in FACS buffer at a concentration of 0.5 μg/mL for 1 h. After incubation, unbound antibodies were removed by three washes in FACS buffer and centrifugation at 450× g for 10 min. Samples were analyzed by collecting a minimum of 5,000 events using a BD LSR Fortessa (Becton Dickinson) cell analyzer equipped with BD FACS Diva software (Fig. 1D).

### Particle and cell flow experiments

3 × 10^5^ HUVEC cells were seeded on fibronectin-coated flow cells (0.4 Ibidi μ-slide; IBIDI, Planegg/Martinsried, Germany) in media with or without TNFα (25 ng/mL). Twenty-four hours later, 3 × 10^7^ NPS, or LLV, or 3 × 10^5^Jurkat cells (indicated as “leukocytes” in the Results section) were introduced into the flow cell at a rate of 0.1 dyn/cm^2^ for 30 min. Cells were subsequently fixed and prepared for microscopy as described below (Fig. 4C). We used the same conditions for live microscopy experiments. Intracellular Ca^2+^ levels were monitored using Fluo-3/AM, Calcium Indicator (Life Technologies) according to the vendor’s specifications (Fig. 3C).

### Immunofluorescence

After particle flow (see above), cells were fixed with 4% PFA, washed twice with PBS 1%-BSA 0.2%-Triton for 5 min. Before and after hybridization with the primary antibody (anti-VE-cadherin Ab-33168 “Abcam” - Cambridge, UK) cells were washed with PBS 1%-BSA. Secondary antibody hybridization was performed using Alexa Fluor® 488 labeled anti-rabbit (Thermo Scientific, Waltham, MA, USA). Nuclei were stained using DAPI (Figs 3A and 4C). Images were taken using an Inverted Nikon FLUO-Scope (Nikon, Tokyo, Japan). Data were analyzed with Nikon software ND2. For particle immunofluorescence, samples were prepared as described above for flow cytometry. After particle conjugation with antibody, 10^5^ particles were seeded on an 8-well Nunc® Lab-Tek® Chamber Slide™ (Thermo Scientific). Images were acquired with a Nikon A1 confocal imaging system and analyzed with Nikon NIS Elements software (Nikon).

### Western Blot Analysis

Whole cell lysate from Jurkat cells and HUVECs were used in this study. Cells were washed with PBS twice and collected by centrifugation at 125 × g for 10 min. Cells were resuspended in RIPA buffer (5 mM Tris-HCl (pH 7.6), 150 mM NaCl, 1% NP-40, 1% sodium deoxycholate, 0.1% SDS) supplemented with PMSF, Protease and Phosphatase inhibitor cocktail (Thermo Scientific), according to the vendor’s indications. Extracts were kept on ice, and the samples were flowed through a needle to increase protein yield. Protein extracts were centrifuged at 14,000 × g for 15 min to separate the proteins (supernatant) from the cellular debris (pellet). The concentration of protein in the extracts was measured with a Bradford protein assay. 30 μg of total protein extracts were loaded onto a 10% Mini-PROTEAN® TGX™ Precast Gel (BioRad, Hercules, CA, USA). For particle characterization, 30 μg of Jukat cell extract, 1 million LLV and NPS were loaded onto the gel (Fig. 1C) (LLV were coated using 150 μg of cell membrane proteins). For phosphorylated VE-cadherin, VE-cadherin, 1.5 × 10^6^ HUVECs were plated onto fibronectin-coated 10 mm cell culture dishes with or without media containing TNFα (25 ng/mL). Then, 90 × 10^7^ NPS, J774-LLV and Jurkat-LLV, 1.5 × 10^6^ Jurkat cells were added to the media for 15 min (Fig. 4A). The proteins from the gels were blotted on a PVDF membrane using a BioRad Trans-Blot® Turbo™ Transfer Starter System according to the vendor’s instructions. After 2 h blocking solution (Tris-Buffered Saline 0.1% Tween 20, 5% Blotting Grade Blocker Non Fat Dry Milk; BioRad) the membranes were hybridized with primary antibody [Anti VE-cadherin phospho ab22775 from: Abcam, Anti-VE-cadherin ab33168 from: Abcam, and Anti-GAPDH sc-137179 from Santa Cruz Biotechnology, Dallas, TX, USA)]. Antibodies were used according to vendor’s indications. For detection we used the Pierce ECL Western Blotting Substrate (Pierce, Waltham, MA, USA) and the BioRadChemiDoc™ MP System (BioRad).

### Animal Care

Animal studies were conducted in accordance with the guidelines of the Animal Welfare Act and the Guide for the Care and Use of Laboratory Animals following approved protocols established by The University of Texas M.D. Anderson Cancer Center’s Institutional Animal Care and Use Committee. Female 8–9 week old BALB/c mice were purchased from Charles River Laboratories (Boston, MA, USA) and maintained using previously established protocols^8^. Mouse breast cancer tumors were established using a single injection of 2 × 10^5^ 4T1-luc2-tdTomato Bioware® Ultra Red from PerkinElmer (Waltham, MA, USA) into the mammary fat pad. At pre-determined times, animals’ images were acquired using an IVIS 200 imaging system (Perkin Elmer). Tumors were determined as established upon reaching a size of 0.8 cm^3^.

### Intravital Microscopy Imaging

Animals were anesthetized using isoflurane. After removing hair, the tumor mass was exposed under the microscope. 40 μl of FITC 70 KDa dextran solution was injected endovenously to maximize the definition and resolution of the vascular bed. In 3 animals per experimental group, 1 billion NPS or LLV were injected. Dextran and particles were systemically administered through the retro-orbital venous plexus. To analyze tumoritropic accumulation and binding stability, the animals were imaged after 1 h and monitored for 2 hours after particle injection. To evaluate the dextran extravasation time course, we used 3 mice per point and analyzed 5 different fields per mouse (Fig. 6). We filmed and collected images for 45 min after particle injection. Dextran diffusion images were taken from the last frames of the IVM movies. Fluorescence intensity was quantified using ND2 software from Nikon.

### Statistical Analysis

Statistical analyses were calculated using Prism GraphPad v. 6.0. All experiments were the result of a minimum of three biological replicates unless stated. Statistics for the immunofluorescence intensity of VE-cadherin expression was analyzed using a one-way ANOVA with a Turkey post-test comparing means. Statistics for dextran extravasation was analyzed using a two-way ANOVA with a Bonferroni post-test.

## Additional Information

**How to cite this article**: Palomba, R. *et al*. Biomimetic carriers mimicking leukocyte plasma membrane to increase tumor vasculature permeability. *Sci. Rep.* **6**, 34422; doi: 10.1038/srep34422 (2016).

## Supplementary Material

Supplementary Information
