# Supplementary material for: Biomimetic carriers mimicking leukocyte plasma membrane to increase tumor vasculature permeability
Source: Sci Rep. 2016 Oct 5;6:34422. doi: 10.1038/srep34422 (PMC5050497; doi:10.1038/srep34422)
Supplement: Supplementary Information [file srep34422-s1.pdf]

Supplementary Information

Biomimetic carriers mimicking leukocyte plasma membrane to increase tumor vasculature permeability

R. Palomba, A. Parodi, M. Evangelopoulos, S. Acciardo, C. Corbo, E. de Rosa, I. K. Yazdi, S. Scaria, R. Molinaro, N.E. Toledano Furman, J. You, M. Ferrari, F. Salvatore and E. Tasciotti

The PDF file includes twelve supporting figures and related materials and methods.

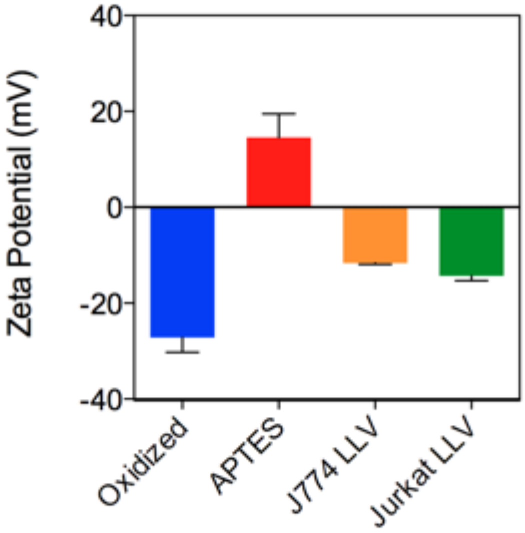

Supplementary Figure S1. Zeta potential analysis of particles before surface modification (Oxidized) and following modification with APTES and cellular membrane coating (J774 and Jurkat LLV).

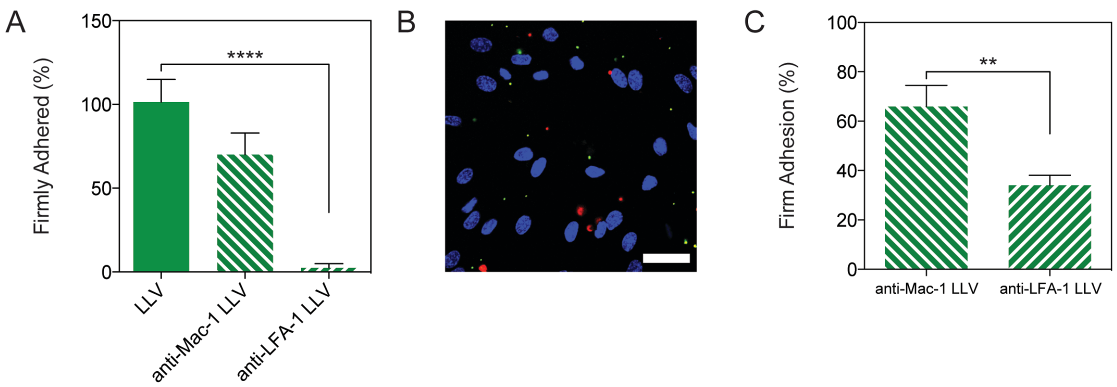

Supplementary Figure S2. (A) Adhesion of LLV and LLV blocked for Mac-1 (anti-Mac-1 LLV) and LFA-1 (anti-LFA-1 LLV) onto endothelial cells under flow conditions. The data were normalized against LLV. (B) Representative fluorescent image of endothelial cells simultaneously treated with LLV blocked for Mac-1 (anti-Mac-1 LLV, green) and LFA-1 (anti-LFA-1 LLV, red) under flow. The nucleus of endothelial cells was stained with DAPI (blue). (C) Relative quantitative analysis of fluorescent images normalized to total amount of adhered particles. Scale bar = 50 µm. The data are plotted as the mean ± s.e.m. ** $p < 0.01$; **** $p < 0.0001$.

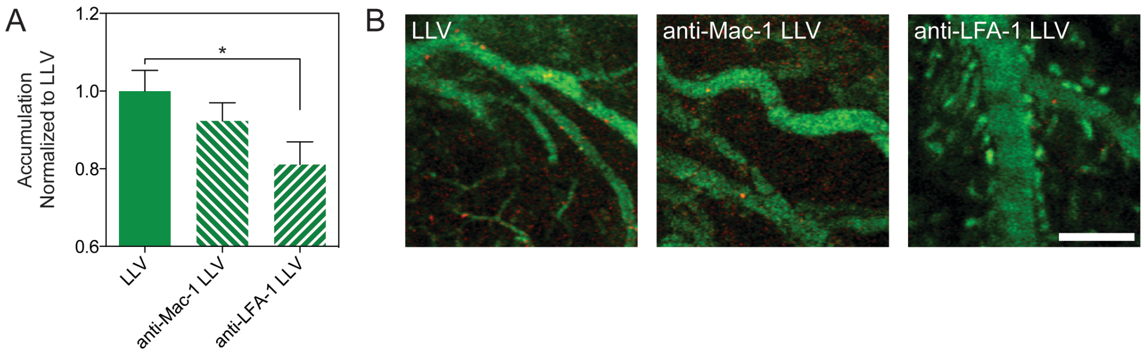

Supplementary Figure S3. (A) Quantification of tumor accumulation of LLV and LLV blocked for Mac-1 (anti-Mac-1 LLV) or LFA-1 (anti-LFA-1 LLV). (B) Representative picture of LLV, and Mac-1 and LFA-1 blocked LLV. All the particles were stained in red, while tumor blood vasculature was labelled in green. Scale bar = 100 µm. The data are plotted as the mean ± s.e.m. * $p < 0.05$.

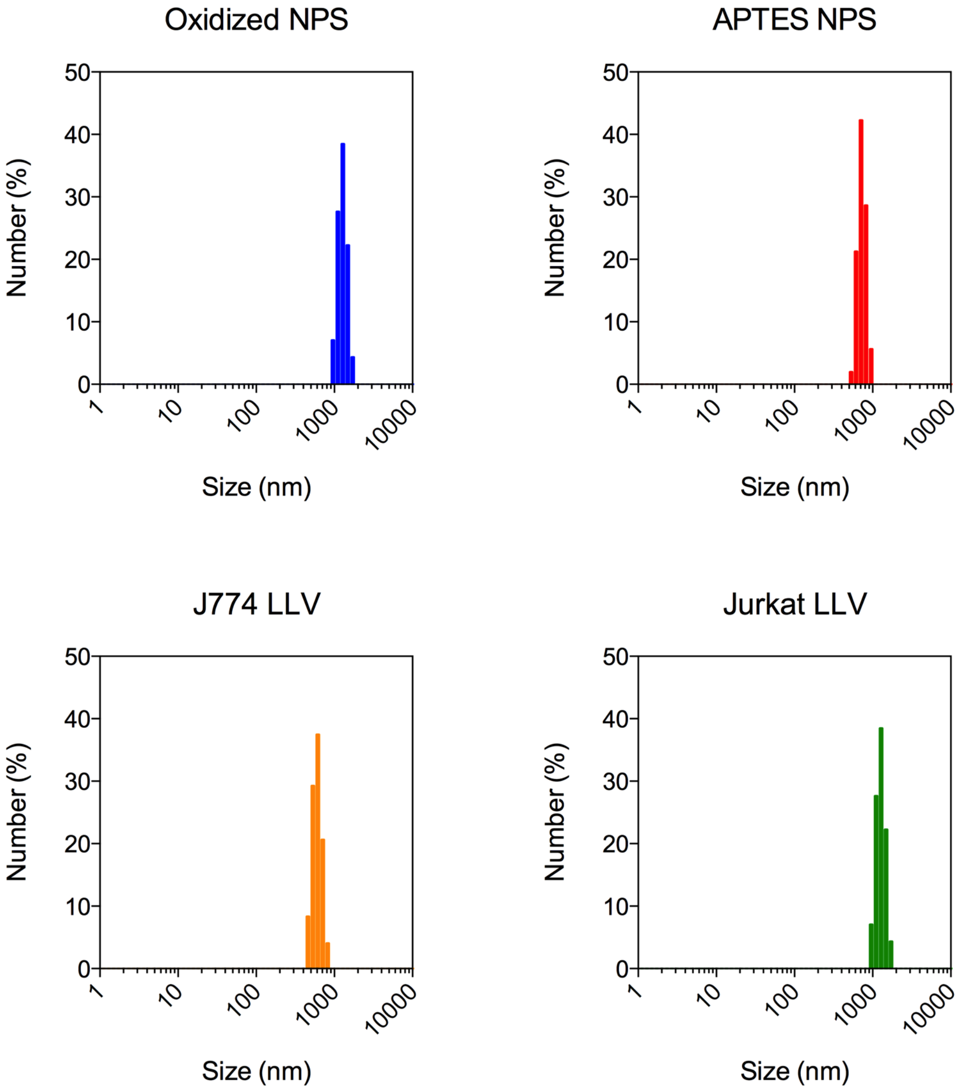

Supplementary Figure S4. Dynamic light scattering analysis of particles following modification with APTES and cellular membrane coating.

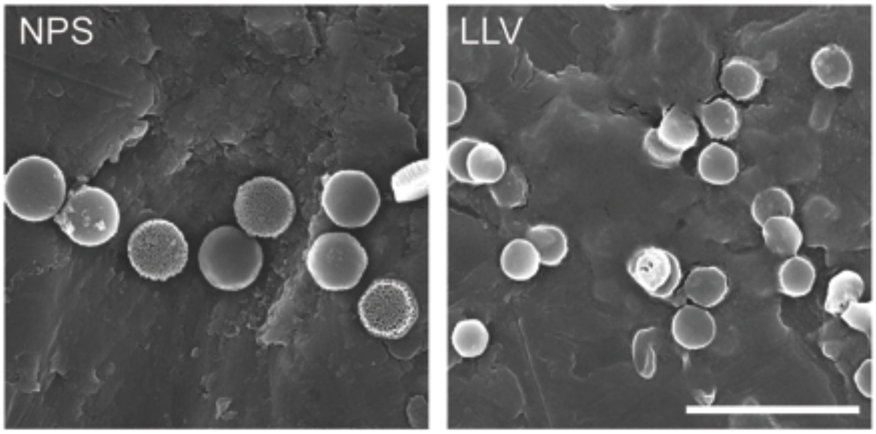

Supplementary Figure S5. SEM image of particles before (NPS) and after (LLV) cell membrane functionalization. Scale bar = 5 μm.

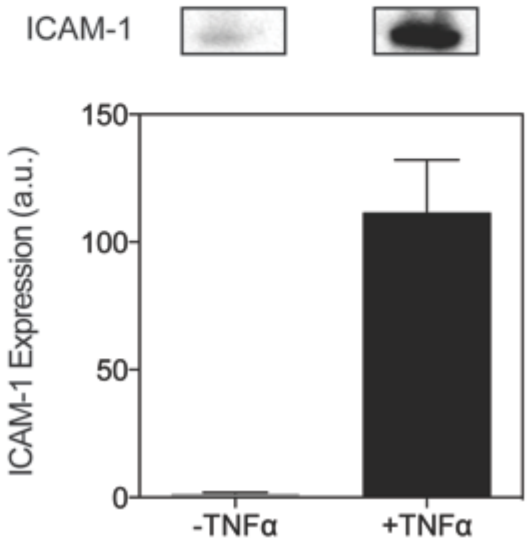

Supplementary Figure S6. Real-time PCR and western blot analysis of ICAM-1 expression in HUVEC with and without TNFα activation. The data are plotted as the mean ± s.d.

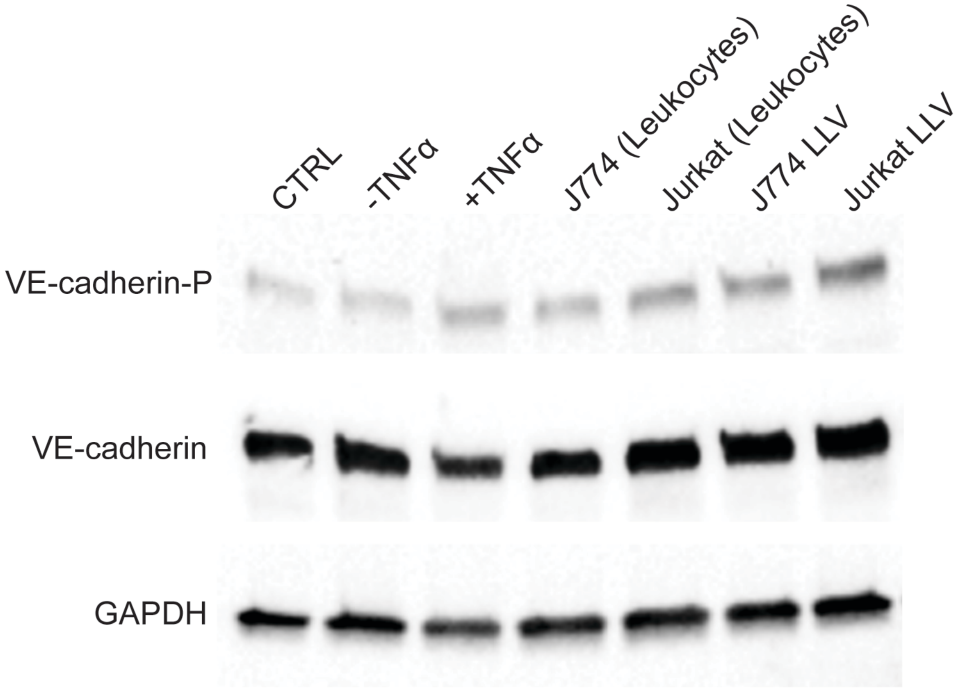

Supplementary Figure S7. Expanded Western Blot image of Fig. 4A depicting HUVEC treated with several treatment groups. Image includes treatment groups -TNFα, +TNFα, J774 (Leukocytes), and J774 LLV that were not included in Fig. 4A.

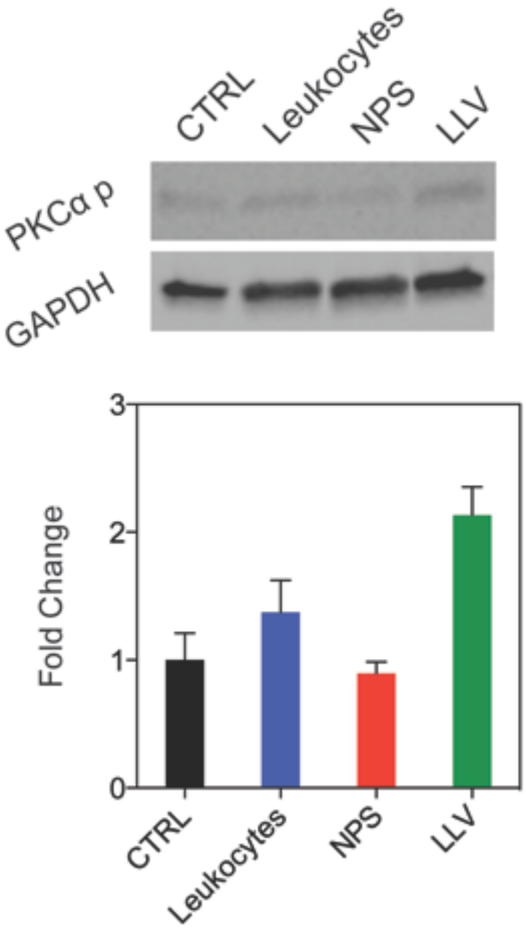

Supplementary Figure S8. Western blot analysis of PKCα phosphorylated protein in TNFα activated HUVEC following LLV treatment. The data are plotted as the mean ± s.d.

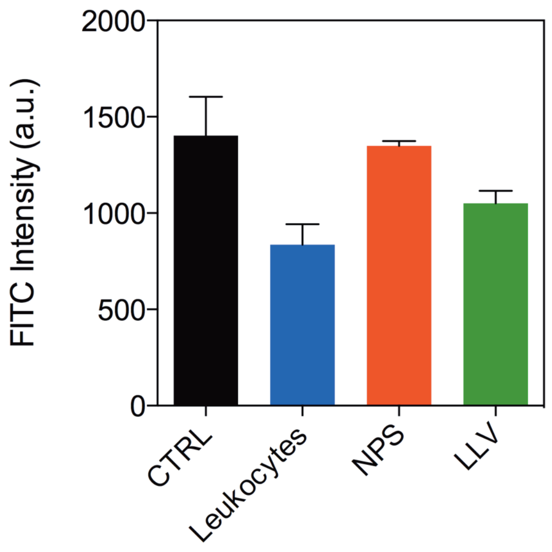

Supplementary Figure S9. Quantitative analysis of VE-cadherin expression on the cell border of non-TNFα-activated HUVEC treated with a flow of leukocytes or particles. Data were obtained by immunofluorescence. Fluorescence intensity was measured along the perimeters of 15 HUVEC per experimental point. The analysis was performed on untreated HUVEC (CTRL) and on HUVEC treated with Jurkat cells (Leukocytes), uncoated particles (NPS), and Jurkat coated particles (LLV). The data are plotted as the mean ± s.d.

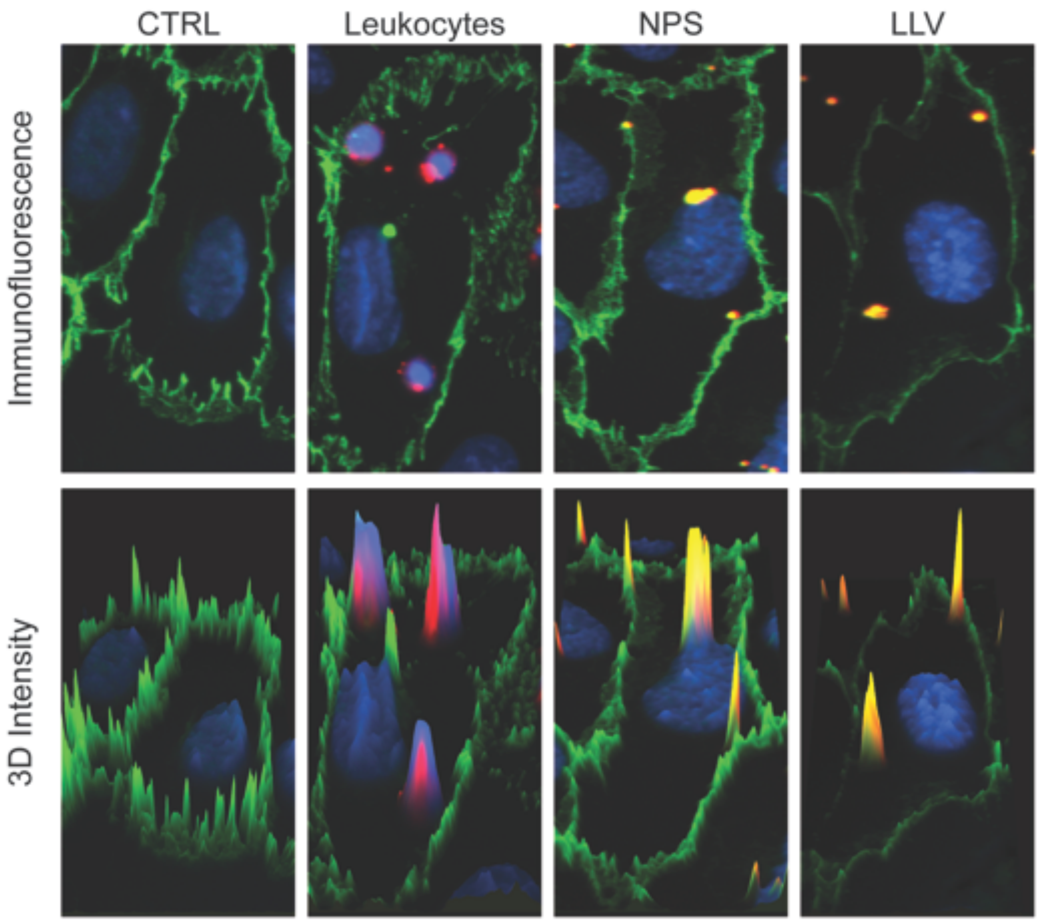

Supplementary Figure S10. Immunofluorescence images and tri-dimensional fluorescence intensity profile (3D Intensity) performed on single non-TNFα-activated HUVEC. The analysis was performed on untreated HUVEC (CTRL) and on HUVEC treated with Jurkat cells (Leukocytes), uncoated particles (NPS), and coated particles (LLV).

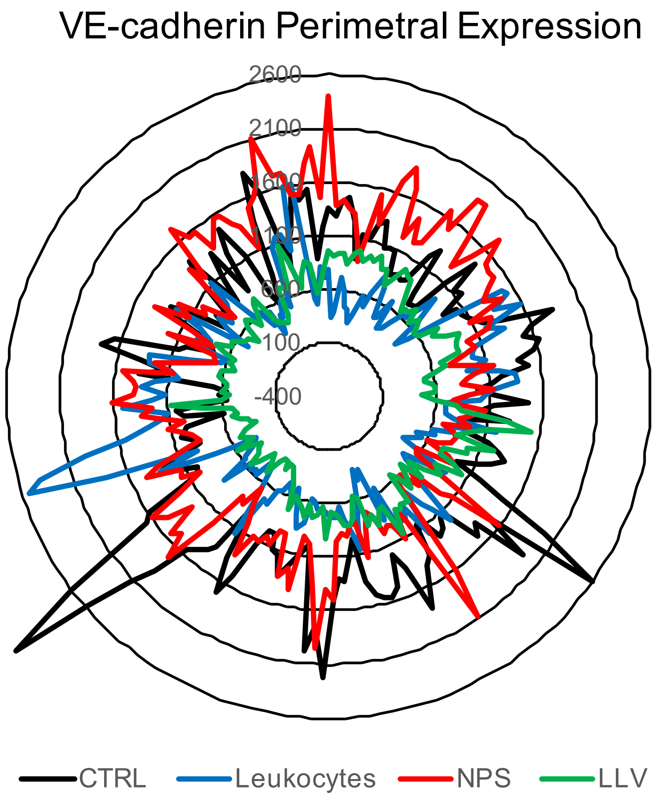

Supplementary Figure S11. Intensity profiles of the cell perimeter of single non-TNFα-activated HUVEC plotted in polar coordinates.

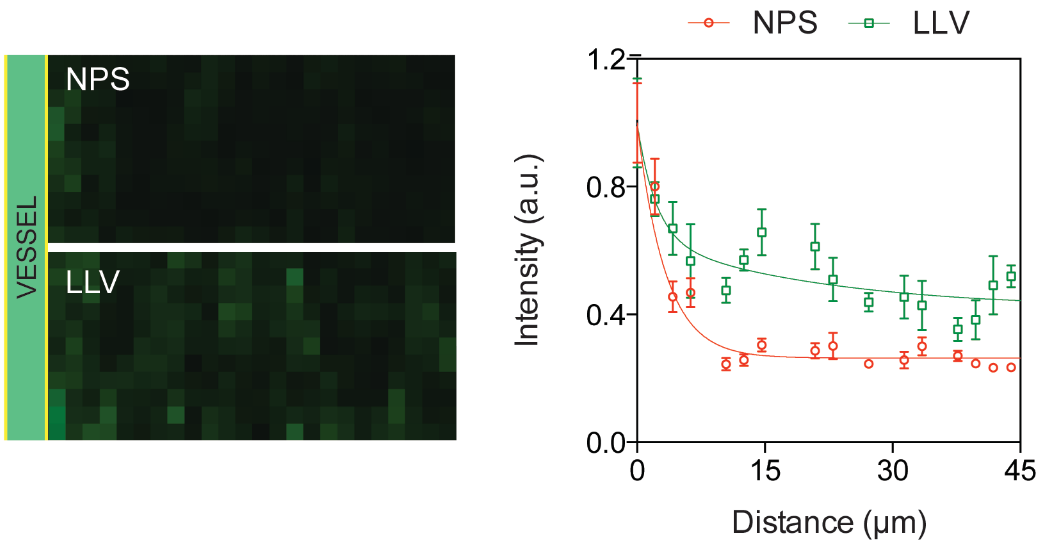

Supplementary Figure S12. Analysis of the diffusion of 70 kDa dextran in the tumor microenvironment. The graph represents the relative fluorescence intensity at different distances from the vessel wall. The data are plotted as the mean ± s.e.m.

Materials and Methods:

Cell Culture. HUVEC were cultured in endothelial cell growth media (EBM-2 Basal Medium) supplemented with EGM™-2 SingleQuots® (Lonza Group Ltd, Basel, Switzerland). All culturing equipment (flasks, slides, plates) were coated with human fibronectin (Becton Dickinson, Houston, TX, USA) 50 µg/ml in PBS. J774 cells were cultured in Dulbecco's modified eagle medium from Lonza supplemented with 10% fetal bovine serum (FBS) and 1% penicillin/streptomycin. Jurkat cells were cultured in Roswell Park Memorial Institute medium (RPMI) from Lonza supplemented with 10% FBS and 1% penicillin/streptomycin. 4T1-luc2-td Tomato Bioware® Ultra Red Light Producing Cell Line mouse mammary adenocarcinoma cells (Perkin Elmer, Waltham, MA, USA) were cultured in RPMI supplemented with 10% FBS and 1% penicillin/streptomycin (Lonza). All cultures were grown at 37°C and 5% CO2.

Scanning electron microscopy. NPS particles and LLV were prepared for SEM by spotting 10^6 particles onto an aluminum stub after five washes in MilliQ water. Samples were left to dry overnight in a well-ventilated area and then sputter-coated with a 5-nm layer of gold. Images were acquired using an FEI quanta 400 ESEM FEG instrument (Hillsboro, OR, USA) equipped with an Everhart-Thornley detector*,* secondary electron detector (Fig. S5).

ICAM-1 Expression. The expression of Intracellular adhesion molecule 1 (ICAM-1) was investigated by western blot. Total protein extract from HUVECs were analyzed using the anti-ICAM-1 (G-5) sc-8439 antibody (Santa Cruz Biotechnology, Dallas, TX, USA). Gene expression was analysed using StepOnePlus™ Real-Time PCR (Applied Biosystems®, Foster City, CA, USA). Total mRNA was extracted from fully confluent 10mm cell culture dish of HUVECs treated or not with TNFα (25ng/mL) using TRIzol® (Life Technologies, Carlsbad, CA, USA). Retro-transcription was performed using SuperScript® II Reverse Transcriptase (Life Technologies). Real time analysis was performed using TaqMan® Fast Advanced Master Mix (Life Technologies) and the specific human ICAM1 probe (Hs00164932_m1 Life Technologies). All reagents were used according to the vendor's indications.

PKCα Western Blot. The analysis was conducted at the same conditions described in main text methods. The antibody used were the following: Anti-PKCα phospho ab32502 from Abcam and Anti-GAPDH sc-137179 from (Santa Cruz Biotechnology Dallas, TX, USA).

Evaluation of LLV mechanism of adhesion. To identify the role of LFA-1 and Mac-1 molecules responsible for the LLV targeting mechanism, we investigated both *in vitro* and *in vivo*, particle adhesion to inflamed endothelium through alternative blockage of these two specific biomarkers (i.e., LFA-1 and MAC-1) using previously established protocols.[1] Briefly, 1 billion LLV were diluted in FACS Buffer (PBS, 1% BSA) and incubated separately with anti-Mac-1 and anti-LFA-1 (2.5 µg/ml) antibodies for 1 h at room temperature. Samples were next purified from unbound antibodies by three washes in 150 mM NaCl centrifugation at 450 × g for 10 min at 4°C and then tested as previously described.[1]

Statistical Analysis. Statistical analyses were calculated using Prism GraphPad v. 6.0. All experiments were the result of a minimum of three biological replicates unless stated. Statistics for the adhesion of particles under shear stress were analyzed using a one-way ANOVA with a Bonferroni post-test comparing means. Statistical analysis for particles co-administered under shear stress were analyzed using a *t*-Test. Statistics for dextran extravasation was analyzed using a nonlinear regression curve fitted with a two phase decay with constraints of Y0 set at 1.0.

References

1 Parodi, A. *et al.* Synthetic nanoparticles functionalized with biomimetic leukocyte membranes possess cell-like functions. *Nature nanotechnology* **8**, 61-68, doi:10.1038/nnano.2012.212 (2013).
